# Prediction of HIV drug resistance based on the 3D protein structure: Proposal of molecular field mapping

**DOI:** 10.1371/journal.pone.0255693

**Published:** 2021-08-04

**Authors:** Ryosaku Ota, Kanako So, Masahiro Tsuda, Yuriko Higuchi, Fumiyoshi Yamashita

**Affiliations:** 1 Department of Drug Delivery Research, Graduate School of Pharmaceutical Sciences, Kyoto University, Kyoto, Japan; 2 Department of Applied Pharmaceutics and Pharmacokinetics, Graduate School of Pharmaceutical Sciences, Kyoto University, Kyoto, Japan; University of Colorado Denver Skaggs School of Pharmacy and Pharmaceutical Sciences, UNITED STATES

## Abstract

A method for predicting HIV drug resistance by using genotypes would greatly assist in selecting appropriate combinations of antiviral drugs. Models reported previously have had two major problems: lack of information on the 3D protein structure and processing of incomplete sequencing data in the modeling procedure. We propose obtaining the 3D structural information of viral proteins by using homology modeling and molecular field mapping, instead of just their primary amino acid sequences. The molecular field potential parameters reflect the physicochemical characteristics associated with the 3D structure of the proteins. We also introduce the Bayesian conditional mutual information theory to estimate the probabilities of occurrence of all possible protein candidates from an incomplete sequencing sample. This approach allows for the effective use of uncertain information for the modeling process. We applied these data analysis techniques to the HIV-1 protease inhibitor dataset and developed drug resistance prediction models with reasonable performance.

## Introduction

Drug-resistant viruses have a significant impact on the prognosis of HIV infections [1, 2]. Predicting drug resistance from their genotypes would allow the selection of appropriate drugs for efficient treatment. The development of such prediction models has been actively promoted [3–7], along with growing databases, such as the Stanford HIV Drug Resistance Database, which collects protein information and evaluates the resistance of drug-resistant viruses [4, 8].

These prediction models include classification or regression models by using various machine learning methods (support vector machine, deep learning, etc.). The models are, unexceptionally, based on the primary sequence of a protein, which is converted into numerical descriptors by means of one-hot encoding or similar techniques and used as predictive variables. Margaret et al. [3] developed a classification model with a high accuracy of approximately 0.9 by using a deep learning technique and detected mutations responsible for drug resistance. Geno2pheno, developed by Niko et al. [4], addressed the regression problem based on a support vector regression (SVR) method and provided a determination coefficient of approximately 0.7. These models are available on websites to easily predict the drug resistance profiles of viral variants.

However, the proposed models have two problems. First, their models are based on the primary protein structure, and therefore lacking information on the 3D structure. Protein function is derived from the tertiary structure, as is commonly understood in structural bioinformatics [9], and it is conceivable that the 3D structure is closely related to drug resistance. Second, there has been a problem in processing incomplete sequencing samples during the modeling procedure. Incomplete sequencing provides multiple candidates at an amino acid position. The authors have views on whether to disregard these data samples [3] or list all possible combinations for modeling use [6, 7]. The former might lead to limited learning with smaller sample sizes, while the latter might overwhelm a model with uncertain data.

With these issues in mind, we propose a novel technique to model drug resistance of HIV. The structural information of HIV-1 variants was encoded as 3D molecular field parameters by conducting homology modeling, structural alignment, and molecular field mapping. The molecular field mapping approach is based on comparative molecular field analysis (CoMFA) [10]. CoMFA is a 3D-QSAR method developed by Cramer et al. that embeds the molecule of interest in a grid lattice and calculates the interaction (i.e., steric and electrostatic potentials) between the probe atoms of each grid point and the molecule. The calculated molecular field parameters are used as features associated with the physical structure of each molecule. CoMFA has been generally used for QSAR analysis of low-molecular weight drugs but, in this study, it was applied to the homologous drug target (i.e., viral protein variants) in a reverse direction. Each possible candidate from incomplete gene sequencing was weighted in the modeling process based on their probabilities of occurrence according to the Bayesian conditional mutual information theory. Partial least squares (PLS) [11], random forest (RF) [12], LightGBM (LGBM) [13], and support vector regression (SVR) [14] were employed to construct the prediction model. We visualized the structural regions with high probability to drug resistance. The flow of the entire analysis was illustrated using several HIV-1 protease inhibitors.

## Methods

### Data collection

HIV-1 drug resistance data, along with the primary structure of HIV-1 protease variants, were obtained from Stanford University’s HIV Drug Resistance Database [8]. Resistance to HIV-1 protease inhibitors was represented by the fold change (FC) increase of the IC50 compared to wild type HIV. In addition, following other papers [3, 15], drug resistance was binarized with a threshold FC of 3.5, where viruses with a higher score were classified as resistant. The number of screening samples was 1059, 665, 1560, 1608, 1373, 1655, 1604, and 766 for atazanavir, darunavir, fosamprenavir, indinavir, lopinavir, nelfinavir, saquinavir, and tipranavir, respectively, of which the number of “complete” sequencing samples was 463, 264, 726, 759, 600, 781, 758, and 302, respectively.

### Treatment of incomplete sequencing samples

**Fig 1:**
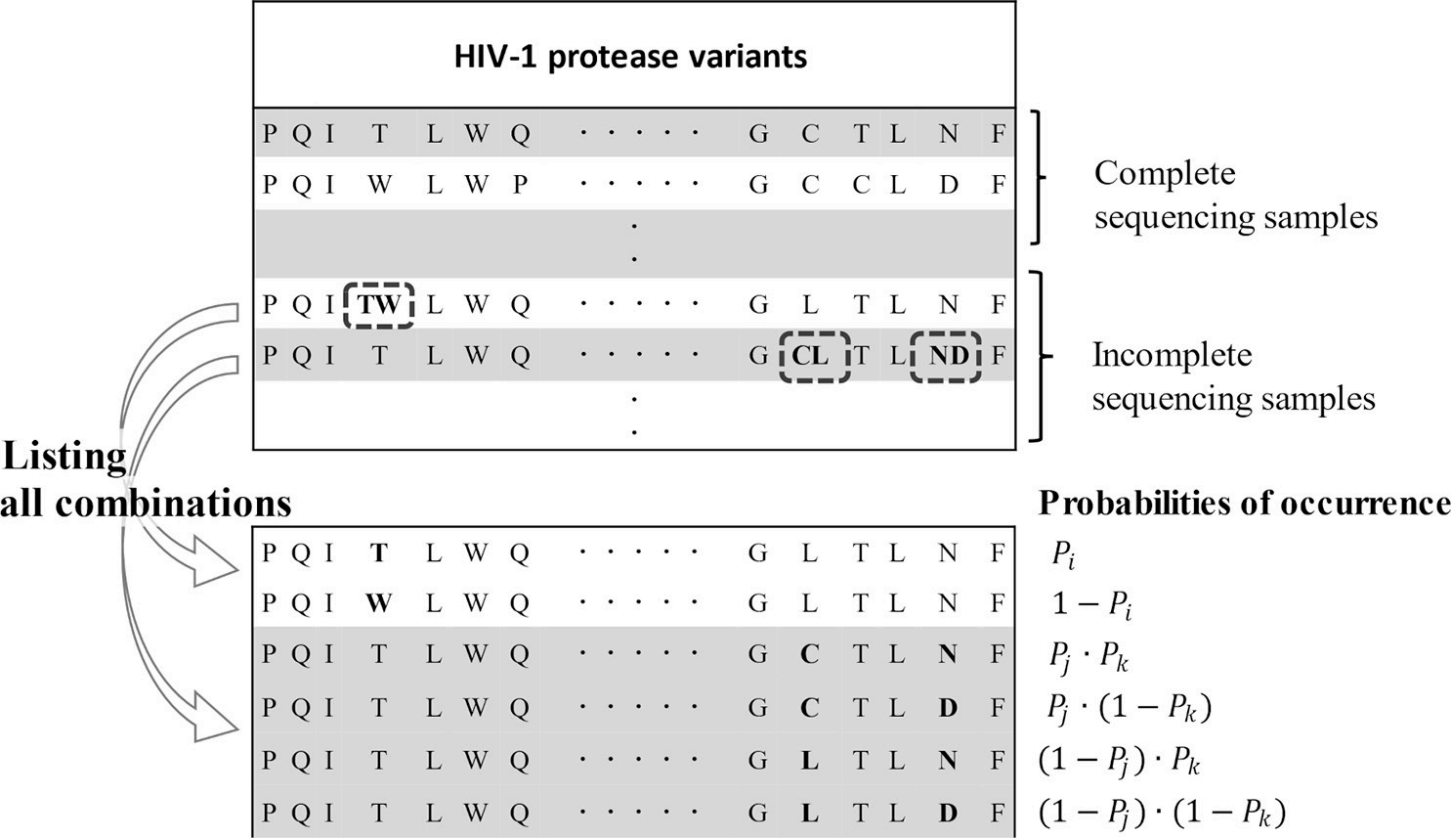
Scheme of treatment of incomplete sequencing samples. All possible combinations were listed from incomplete sequencing samples, and their information was weighted by the conditional probability of occurrence. The conditional probability of occurrence of each amino acid at each position was determined based on amino acid sequences of complete sequencing samples.

The data collection contained incomplete sequencing samples, such that multiple candidates existed at certain amino acid positions. We considered the uncertainty of all possible combinations based on their probabilities of occurrence (Fig 1).

The conditional probability of occurrence was calculated for each of the multiple candidates at position *i*, given the type of amino acids at the *m* positions. This idea is based on the concept of population genetics, in which viruses prone to drug resistance have a similar genetic sequence [16].

We consider the type of amino acids as a random variable and let *X_i_*∈(*A,R,N,⋯,Y*) be the *i*-th random variable in the amino acid sequence. According to the marginal probability density function *p*(*X_i_*) and the simultaneous probability distribution function *p*(*X_i_,X_j_*), information entropy *H*(*X_i_*), mutual information content *I*(*X_i_,X_j_*), and normalized mutual information *NMI*(*X_i_,X_j_*) [17] are expressed as follows:

$$H(X_{i})=\sum_{x_{i}\in X_{i}}(-p(x_{i}))∙\log\;p(x_{i})$$ (1)

$$I(X_{i},X_{j})=\sum_{x_{i}\in X_{i}}\sum_{x_{j}\in X_{j}}p(x_{i},x_{j})∙\log\frac{p(x_{i},x_{j})}{p(x_{i})∙p(x_{j})}$$ (2)

$$NMI(X_{i},X_{j})=\frac{I(X_{i},X_{j})}{\sqrt{H(X_{i})∙H(X_{j})}}\;(0\leq NMI(X_{i},X_{j})\leq 1)$$ (3)

The NMIs for all pairs were determined from a collection of complete sequencing samples. A permutation test [18] was performed to determine the statistical significance (P<0.05) for the NMIs. For each *X_i_*, a set of statistically relevant positions in the sequence (***X***_***m*,*i***_) was determined. ***X***_***m*,*i***_ was limited to the top 10 if the length was greater than 10. Finally, the conditional probability *p*(*X_i_* = *x_i_*|***X_m,i_*** = ***x_m_***) was calculated.

### Calculation of molecular field potentials

Modeller 9.20 [19] was used for homology modeling [20]. The template candidates for each HIV-1 protease inhibitor are summarized in S1 Table. The crystal structures of HIV-1 protease proven to bind to the drugs were obtained from the PDB database and used as a template. In addition, mufft v7.427 [21] was used to align the primary sequence with the templates. For each protease variant, the template with the highest percent sequence identity (PID) was used for automated homology modeling using Modeller [22]. The atomic charge of each atom of the proteases was calculated using PDB2PQR version 2.1.1 [23].

Before the molecular field analysis, all homology-modeled protease variants were superimposed on one another [24–35]. The molecular field parameters (i.e., steric and electrostatic potentials) of each variant were calculated using a method similar to that described by Cramer et al. [10]. The protease structure was embedded in a 2 Å spacing lattice. A probe atom with the van der Waals properties of sp^3^ carbon and a charge of +1 was placed at each grid point. The steric and electrostatic interaction energies in the sum between the probe atom and each atom of the protease variants were calculated as the van der Waals [36] and Coulomb’s potentials [37], respectively.

### Construction of prediction models

Data were randomly divided into two parts: 80% for training and 20% for external evaluation. The feature extraction and hyperparameter selection described later were adopted for the training data. A prediction model was developed using PLS, RF, LGBM, and SVR with molecular field parameters as predictors and drug resistance as the output. PLS was implemented in Python 3.7 (S1 File). LGBM model were constructed using LightGBM 2.3.0, while RF and SVR using scikit-learn 0.23.1.

### Feature selection

Feature selection was conducted using only complete sequencing samples. The first concern was that the calculation of the molecular field parameters diverged when the distance between the probe and target atoms was too close. In the first feature selection stage, the lower and upper limits were set to the 5th percentile of the dataset (*cutoff*_5%_) and the 95th percentile of the dataset (*cutoff*_95%_), respectively. In addition, the molecular field parameters with a heavily biased distribution of potential energies within the samples were removed from modeling, where the absolute skewness was greater than 2.5. The molecular field parameters with a standard deviation of 2 kcal/mol or less were also removed [38].

In the secondary feature selection stage, the molecular field parameter dataset was subjected to machine learning-based recursive feature elimination [39]. The importance of the molecular field parameters as a feature of modeling was estimated using the linear model (scikit-learn) of SVR. Feature selection for the steric and electrostatic molecular field parameters was performed independently. For the steric potential, the unimportant data was removed recursively two at a time, until the remaining number reached one-half of the first stage. On the other hand, in the case of the electrostatic potential, the data were removed recursively ten at a time, until the remaining number reached one-eighth.

### Machine learning models

The loss function and evaluation function (weighted determination coefficients) of the regression model are defined as follows:

$$Loss\;function=\sum \sum p_{ij}{∙(y_{obs,i}-y_{pred,ij})}^{2}$$

$$Evaluation\;function\;(R^{2})=1-\frac{\sum \sum p_{ij}{∙(y_{obs,i}-y_{pred,ij})}^{2}}{\sum {\left(y_{obs,i}-{\bar{y}}_{obs}\right)}^{2}}.$$

where *y_obs,i_* and ${\bar{y}}_{obs}$ are the observed log_10_ FC of IC_50_ for sample *i* and their average, respectively, and *y_pred,ij_* and *p_ij_* are the log_10_ FC and conditional probability for the *j*-th candidate of sample *i*, respectively.

### Hyperparameter optimization

For each machine learning model, the corresponding hyperparameters were optimized using Optuna version 2.0.0 [40], which implements the tree-structured Parzen estimator (TPE) algorithm. The samples were divided into three groups for 3-fold cross-validation [41]. One group was used for validation data, while the remaining two were used as training data. The training data were preprocessed as described in the previous section and then normalized. The validation data were processed using the conditional parameters used for the training data. Next, each machine learning model was trained with the training data to minimize the loss function and, finally, evaluated with the validation data in terms of predictive *R*^2^. The cross-validation was repeated three times, and the average of the predictive *R*^2^ was used for the evaluation of hyperparameters. The hyperparameters were updated 30 times in Optuna.

### Final evaluation

As mentioned above, 80% of the total data was used for training, and the remaining 20% was used for external evaluation. After preprocessing and normalizing the training data, each regression model was built using the hyperparameters determined during the cross-validation step. Regression models were tested using external data and evaluated by weighted determination coefficients. Setting the threshold of FC at 3.5, the goodness of the classification was evaluated with accuracy, precision, true-positive rate (TPR), true-negative rate (TNR), false-positive rate (FPR), false-negative rate (FNR), area under the ROC curve (AUC), and F1 score [42, 43].

### Visualization of structural importance in drug resistance acquisition

Molecular field-based analysis allows visualization of sites of importance involved in acquisition of drug resistance. It should be noted that spatially neighboring grid points show relatively similar potential energies because the molecular field is continuous. The ability of PLS to construct models considering the collinearity of predictor variables is also useful for detecting regions of high susceptibility to drug resistance. Standardized partial regression coefficients obtained with PLS analysis show the degree of importance of drug resistance acquisition for each spatial coordinate. Upon setting certain thresholds (the 1st and 99th percentiles of the coefficient), a contour map was created in the 3D molecular field. First, grid points remaining after feature extraction were used for PLS modeling for drug resistance and subjected to PLS standardized partial regression coefficients. Next, for all grid points connected by the Delaunay algorithm, the interior points of the line segments of the mesh were searched where the indicated threshold score was given. Finally, a contour map was created by connecting points less than 5 Å apart and creating a new cluster. The figure was generated using Chimera version 1.15 [44].

The degree of agreement of the contour map with known major drug resistance-related amino acid positions (30, 32, 47, 48, 50, 54, 76, 82, 84, 88) [8] was assessed. Firstly, the distance to the nearest contour plane for all α-carbons of the protease was calculated, and their cumulative probability density distribution was obtained. Then, the simultaneous probability for the major drug resistance positions was estimated and compared with the probability for a random selection of the same number of amino acid positions. One hundred thousand random permutations were generated to estimate the ranking for the drug resistance positions.

## Results and discussion

### Sequence data collection and processing

**Fig 2:**
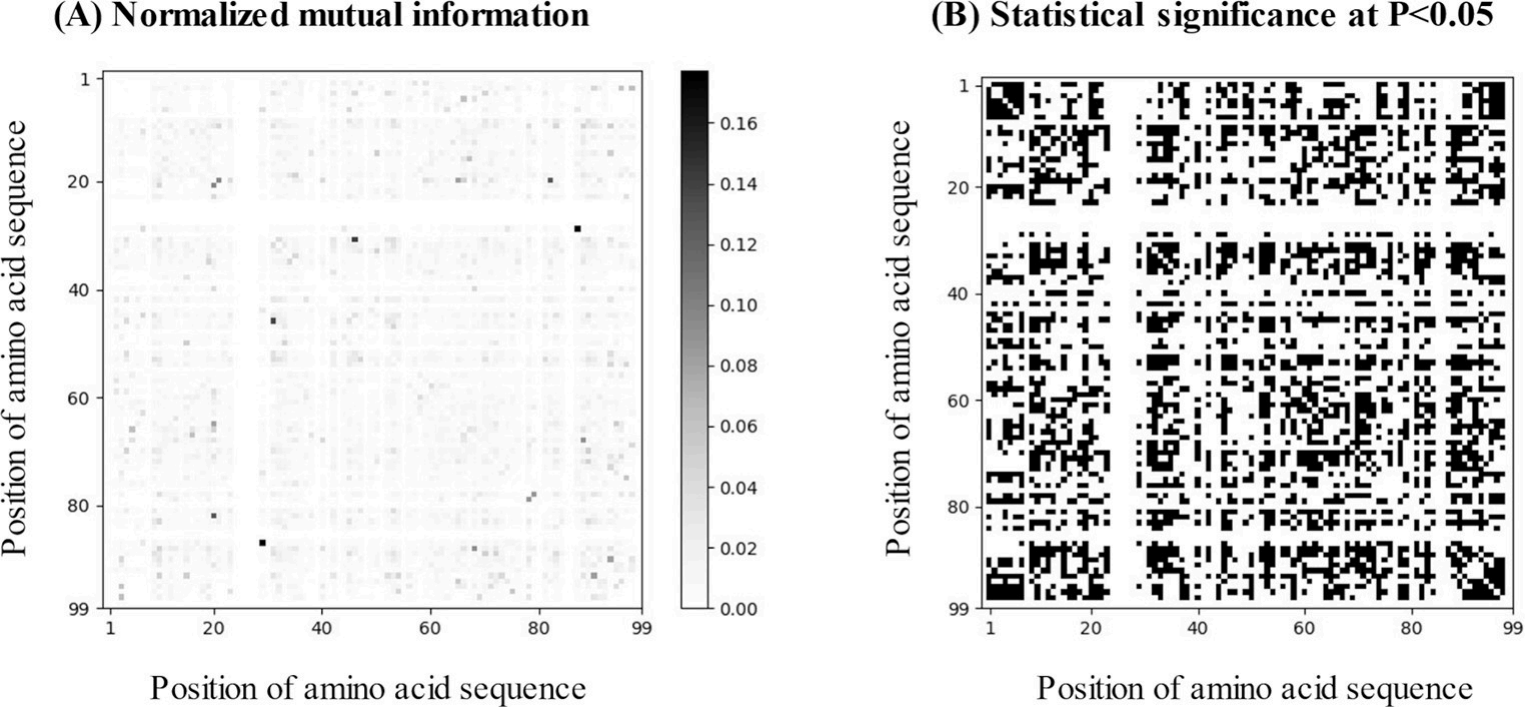
Normalized mutual information on co-occurrence of amino acids between any two positions. (A) Gray-scale image matrix of normalized mutual information (NMI). (b) Statistical significance of NMI determined by permutation test at P<0.05 (black).

Table 1 summarizes the sample sizes of the eight HIV-1 protease inhibitors listed in the database. The sample size varied among the drugs since they were not tested for all viral variants. A considerable number of incomplete sequencing samples were included in the database. Previous researchers have expanded them into all possible combinations of primary sequences [6, 7]. The incompleteness of gene sequencing might be associated with a mixture of viral variants or the detection limit of the Sanger method [45]. It should be noted that a simple listing of combinations would result in unreliable and erroneous information. Indeed, the number of all possible combinations in incomplete sequencing samples was much larger than that of complete sequencing samples. Being aware of the necessity of data weighting, we introduced the conditional probability for each sequence based on the concept of population genetics [16]. Using complete sequencing samples, we calculated the NMI for each pair of different positions in the sequence (Fig 2), performed a permutation test to determine the statistical significance of the NMIs, determined a set of up to 10 statistically relevant positions for each position, and finally obtained conditional probabilities for each occurrence. We weighted each possible combination with its conditional probabilities and estimated a substantial number of samples for each drug (Table 1). As a result, we roughly doubled the information compared to the complete sequencing samples.

**Table 1:** Numbers of sequencing samples listed in the database for each HIV-1 protease inhibitor.

Drug	Number of complete sequencing samples	Incomplete sequencing samples		
		Number of samples	Total sum of all combinations^a^	Practical total sum of all combinations^b^
Atazanavir	463	596	219847	407.97
Darunavir	264	401	210578	275.50
Fosamprenavir	726	834	232626	581.63
Indinavir	759	849	233116	597.86
Lopinavir	600	773	231749	536.13
Nelfinavir	781	874	234104	613.68
Saquinavir	758	846	233304	592.94
Tipranavir	302	464	212140	316.77

a) The total sum of all possible combinations of sequences in each incomplete sequencing sample.

b) The total sum of all possible combinations weighted by their probability of occurrence in each incomplete sequencing sample.

### Calculation of molecular fields and feature selection

The 3D structure of each HIV-1 protease variant was predicted using homology modeling. Since several crystal structures of drug-protease complexes are available for each drug, the homology modeling template of each protease variant was chosen according to the primary sequence similarity. After homology modeling, all variants were superimposed without reforming their 3D structure onto the most common structural template (the bold symbols in S1 Table). The structures were subjected to calculations of the steric and electrostatic molecular fields in the grid lattice. The molecular field parameters represent the structural similarity/dissimilarity of protease variants, which allow comparison from a physicochemical perspective.

**Fig 3:**
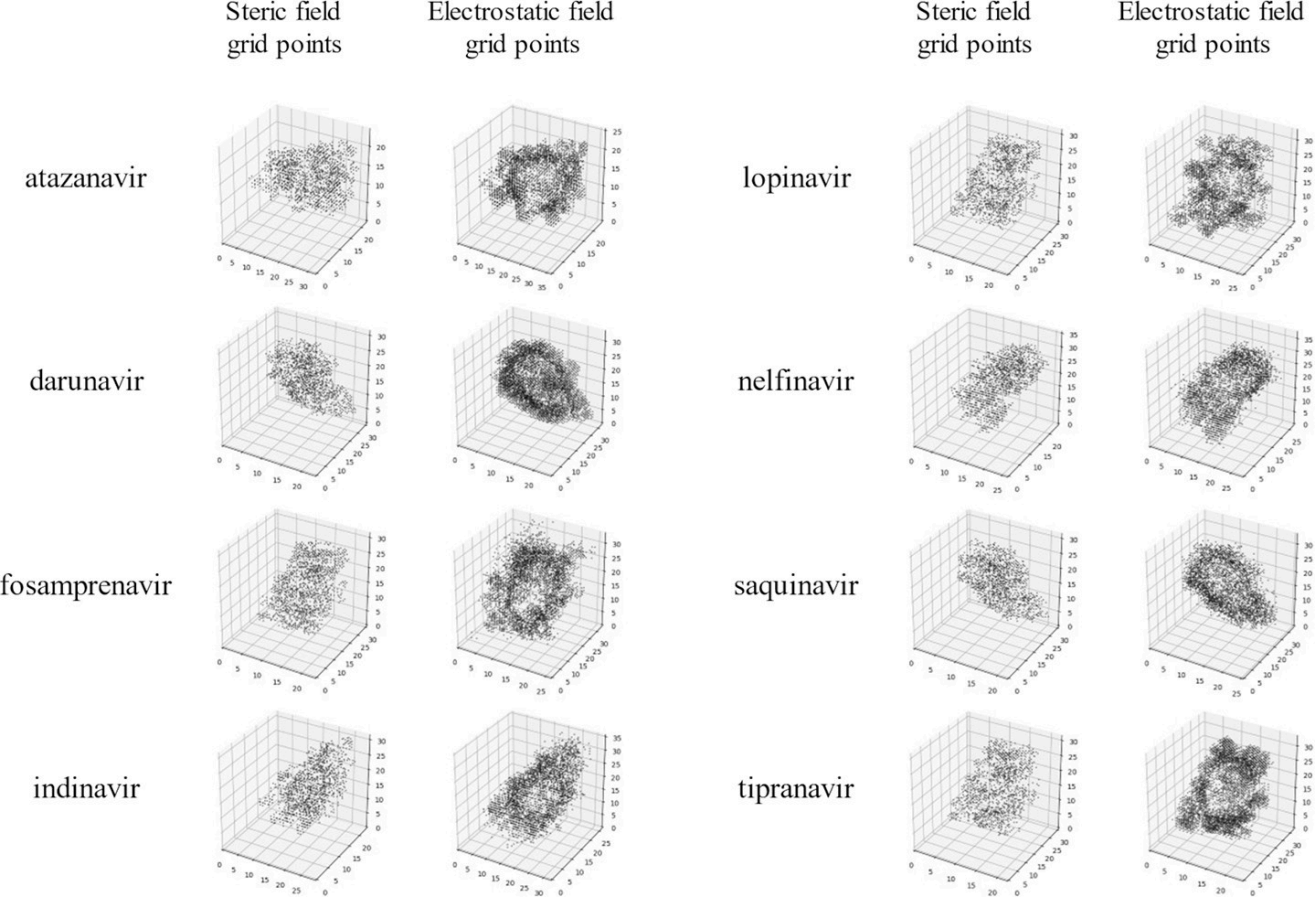
Selected grid points of steric (left) and electrostatic (right) molecular field parameters in the analysis of drug resistance for each HIV-1 protease inhibitor. The grid points were selected by preprocessing and SVR feature selection.

The grid lattice covering the protease variants required 80 Å × 70 Å × 56 Å in size. In the case of setting the grid interval to 2.0 Å, both steric and electrostatic molecular field parameters brought the total number to 60,000. Feature selection were performed to increase the efficiency of machine learning, resulting in a reduction in the number of parameters to approximately 4,000 (S2 Table). Fig 3 shows the coordinates of the extracted features. The coordinates of the remaining molecular field parameters almost reflected the shape of the proteases, suggesting that the features were reasonably extracted.

### Model performance

The data set was divided into training and external test datasets for each drug (S3 Table). PLS, LGBM, RF, and SVR, which are widespread and computationally less-intensive algorithms, were selected to build the prediction models. The ranges of hyperparameters in model optimization are given in S4 Table, and the results are summarized in S5 Table. In PLS, the number of principal components was determined by 3-fold cross-validation (S5 Table). The weighted determination coefficients and 3-fold cross-validated predictive determination coefficients for the training data are summarized in S6 and S7 Tables, respectively. In addition, predictive determination coefficients for the external test dataset that have never been used for training is summarized in Table 2. The scatter plots of observed and predicted log_10_ FC are shown in S1–S8 Figs. The four models did not significantly differ in their prediction accuracy, but LGBM appeared to provide better prediction accuracy in all cases. A comparison of performance between the present and previous models would make sense, even though different or differently preprocessed datasets were analyzed. The present regression model performed better than Geno2pheno (predictive *R*^*2*^ = 0.698) [4]. Geno2pheno has been developed based on a different, smaller dataset than that of the Stanford Drug Resistance Database. Unfortunately, the present model was slightly inferior to the model proposed by Shen et al. (predictive *R*^*2*^ = 0.883) [7]. Both models used the Stanford Drug Resistance Database, listed all possible combinations from each incomplete sequence sample, and gave the same answer label for each. The difference was that we performed the weighting of the data according to their probability of occurrence, resulting in different predictive performances.

**Table 2:** Weighted determination coefficients for prediction in external test dataset (*R*^2^).

Drug	Weighted determination coefficient			
	LGBM^a^	PLS^a^	RF^a^	SVR^a^
Atazanavir	0.792	0.770	0.737	0.759
Darunavir	0.749	0.694	0.711	0.693
Fosamprenavir	0.718	0.667	0.683	0.701
Indinavir	0.830	0.790	0.783	0.806
Lopinavir	0.862	0.810	0.829	0.837
Nelfinavir	0.760	0.743	0.714	0.705
Saquinavir	0.776	0.632	0.748	0.676
Tipranavir	0.513	0.491	0.470	0.488

Abbreviations: LGBM, LightGBM; PLS, partial least squares; RF, random forest; SVR, support vector regression.

The effectiveness of the weighting of the data based on their probability of occurrence was checked using the same training and external test datasets. Two additional types of prediction models were developed by treating the present training data set in a manner equivalent to the models of Geno2pheno and Shen et al.: that is, the former used only the complete sequencing data, and the latter used all combinations without considering their probability of occurrence. The prediction performance was evaluated on the complete sequencing data of the external test dataset. In any case, the best approach was to employ all combinations, considering the probability of occurrence (S8 Table).

Classification models have also been proposed to identify whether viral variants are drug-resistant [3]. For comparison, we used our regression model for classification purposes by defining an FC of 3.5 as a threshold. Table 3 summarizes the results of the external validation assessments. Multiple metrics were used to evaluate the predictive classification performance, which included metrics suitable for both balanced (e.g., accuracy, precision, true positive ratio) and imbalanced (e.g., F1) data. As with the regression purpose, the LGBM model was slightly better than or comparable to the other three machine learning models (Table 3, S9 Table). The accuracy of approximately 0.9 achieved by the models was as high as that of the previously reported classification model [3].

**Table 3:** Goodness of classification by the LGBM model^a^.

Drug	Accuracy	Precision	TPR^b^	TNR^b^	FPR^b^	FNR^b^	AUC^b^	F1 score
Atazanavir	0.914	0.881	0.947	0.883	0.117	0.0533	0.915	0.913
Darunavir	0.921	0.810	0.805	0.951	0.0487	0.195	0.878	0.807
Fosamprenavir	0.896	0.789	0.921	0.885	0.115	0.0794	0.903	0.850
Indinavir	0.924	0.897	0.917	0.928	0.0716	0.0834	0.922	0.907
Lopinavir	0.915	0.895	0.929	0.903	0.0970	0.0711	0.916	0.912
Nelfinavir	0.906	0.888	0.938	0.873	0.127	0.0624	0.905	0.912
Saquinavir	0.909	0.832	0.929	0.898	0.102	0.0712	0.913	0.878
Tipranavir	0.890	0.681	0.567	0.950	0.0497	0.433	0.759	

a) LGBM models were used for classification purposes by defining a fold change (FC) of 3.5 as a threshold.

b) Abbreviations: TPR, true-positive ratio; TNR, true-negative ratio; FPR, false-positive ratio; FNR, false-negative ratio; AUC, area under the ROC curve.

The prediction of drug resistance to tipranavir appeared to be less accurate than that for other drugs. The poor prediction accuracy of tipranavir might be associated partly with an imbalance in number between drug-susceptible and drug-resistant variants (1.0 vs 0.19). As machine learning is a data-dependent analysis, this could simply be a result of chance. However, it might be interesting to note that tipranavir has a slightly different mode of HIV-1 protease inhibition. HIV-1 protease forms a dimer with a catalytic site between the two units [46]. Although most HIV-1 protease inhibitors bind to the catalytic site, tipranavir can also inhibit the formation of the dimer itself [47]. Unfortunately, the present model could not predict the latter activity. Darunavir also possesses the same mechanism as tipranavir [47, 48], but its inhibitory effect was well predicted by the current model. Considering that the primary mechanism of the two drugs is yet to be determined, the limitations of the current model need to be considered.

### Structural factor analysis of drug resistance

The drug resistance of the virus can be attributed to structural changes in viral proteins associated with their mutations. Characterization of the structural effects of protein mutations would be of great use in drug discovery [47]. The proposed models are based on the CoMFA approach, which allows 3D mapping of the degree of importance of the molecular field parameters in the target property/activity [10, 49]. CoMFA has generally been used to analyze and predict the target binding or bioactivity of a series of small-molecule drugs. The implemented PLS algorithm detects the link between the substituents around the core scaffold and the target property. Our model reversed regular CoMFA models, where structurally homologous protein variants that recognize the same drug were superimposed and analyzed. We expected to quantitatively evaluate how much drug resistance was affected by changes in molecular fields associated with the protein mutation.

**Fig 4:**
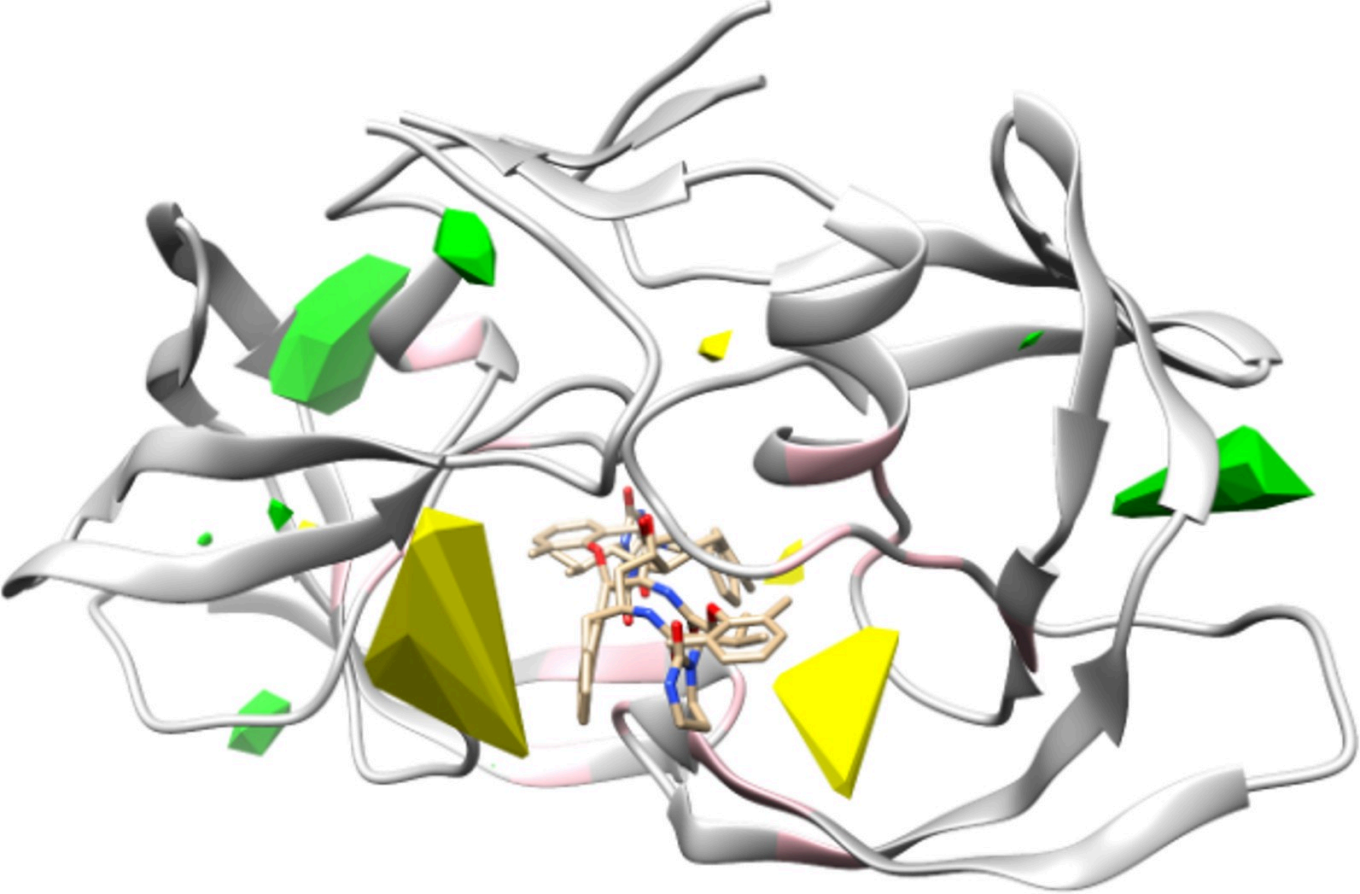
Contour map of steric effects in drug resistance acquisition. Contours were generated based on PLS standardized partial regression coefficients. Yellow and green contours indicate 1^st^ and 99^th^ percentiles of standardized partial regression coefficients, respectively. Steric interaction of the protease with yellow regions negatively affects drug resistance acquisition, whereas green regions show a positive effect. Dimerized wild-type HIV-1 protease (gray ribbon) and lopinavir (wireframe) are shown in the same figure. Pink indicates amino acids involved in drug resistance.

A contour map generated by PLS analysis of molecular fields allows the identification of structural impacts on drug resistance acquisition (Fig 4). Fig 4 also shows the structure of the wild-type protease complexed with lopinavir. Green contours indicate regions in which the steric interaction of the protease increases drug resistance. In contrast, yellow contours indicate regions where the steric interaction lowers drug resistance (i.e., drug resistance increases as the steric interaction decreases). The yellow contours were located near the drug-protein binding interface. The molecular dynamics simulations of Wang et al. [50] revealed that the enlargement of the binding pocket by amino acid mutations weakens the binding of inhibitors. On the other hand, the green contours are located at the peripheries of the protein, where drug-protein interactions are unlikely to occur. We assume that these regions could be a counterpart to the yellow regions. This means that enlargement of the binding pocket might enhance steric interactions at the periphery. The standardized partial regression coefficients of the electrostatic molecular field parameters were much smaller than those of the steric molecular field parameters (data not shown). This suggests that changes in electrostatic interactions due to mutations might be less involved in drug resistance acquisition.

The pink regions of the main chain indicate the position of amino acids in which the mutation confers drug resistance. To investigate how accurately the contour map explains the known drug resistance positions, the distance between the contours and the drug resistance positions was investigated. After estimating the probability distribution of the distance between the α-carbon and the nearest contour, the simultaneous probability for the selection of a set of the major drug resistance positions was calculated and compared to that for a random selection of the same number of amino acid positions. In terms of proximity to the contour planes, the set of the major drug resistance positions ranked in the top 0.316% of 100,000 random permutations. It indicates that known drug resistance-associated amino acids are positioned at the vicinity of the contour maps. Thus, the present 3D-based analysis reasonably represents the structure-activity relationship in the acquisition of drug resistance by viral mutation.

## Conclusion

In this study, we successfully developed a predictive model for HIV drug resistance with reasonable prediction accuracy based on the 3D structure of HIV protease variants. The proposed method can also be applied to predict the resistance to other anti-HIV agents, such as reverse transcriptase inhibitors and integrase inhibitors. It should be noted that the steps of homology modeling and machine learning processes are computationally intensive. Considering that reverse transcriptase and integrase are more than twice as large as protease, it would be difficult to apply the current approach in a limited computational environment.

## Supporting information

S1 Table Template proteins for homology modeling of HIV protease variants. There are several available complex structures with HIV protease variants for each drug. For each protease variant to be subjected to homology modeling, one of the protein structures listed was selected as a template according to the similarity of primary amino acid sequences.
(DOCX)

S2 Table Number of molecular field energies selected as a feature of machine learning. a) Grid sizes were set to embed an overlaid aggregate of all HIV protease variants. b) Numbers of steric and electrostatic potential energies.
(DOCX)

S3 Table Sample size of training and external test datasets for each drug. (DOCX)

S4 Table Ranges of hyperparameters in model optimization. (DOCX)

S5 Table Optimized hyperparameters of LightGBM (LGBM), Random Forest Regression (RF), Support Vector Regression (SVR), and Partial Least Squares (PLS) models for each drug. a) Abbreviations: ATV, atazanavir; DRV, darunavir; FPV, fosamprenavir; IDV, indinavir; LPV, lopinavir; NFV, nelfinavir; SQV, saquinavir; TPV, tipranavir.
(DOCX)

S6 Table Weighted determination coefficients for prediction in training dataset (*R*^2^). (DOCX)

S7 Table Weighted determination coefficients for prediction of 3-fold cross-validation^a)^. a) Data represents mean ± SEM.
(DOCX)

S8 Table Effect of weighting of training data on external prediction in Lopinavir. a) A: All combinations, considering their probability of occurrence, B: All combinations without considering their probability of occurrence, C: Only the complete sequencing data. b) Mean of three runs.
(DOCX)

S9 Table Goodness of classification of Random Forest Regression, Support Vector Regression, and Partial Least Squares models^a)^. a) Goodness of classification was evaluated upon defining the threshold as a fold change of 3.5. b) Abbreviations: TPR, true positive ratio; TNR, true negative ratio; FPR, false positive ratio; FNR, false negative ratio; AUC, area under the ROC curve.
(DOCX)

S1 Fig Scatter plot of observed and predicted drug resistance in atazanavir. (TIF)

S2 Fig Scatter plot of observed and predicted drug resistance in darunavir. (TIF)

S3 Fig Scatter plot of observed and predicted drug resistance in fosamprenavir. (TIF)

S4 Fig Scatter plot of observed and predicted drug resistance in indinavir. (TIF)

S5 Fig Scatter plot of observed and predicted drug resistance in lopinavir. (TIF)

S6 Fig Scatter plot of observed and predicted drug resistance in nelfinavir. (TIF)

S7 Fig Scatter plot of observed and predicted drug resistance in saquinavir. (TIF)

S8 Fig Scatter plot of observed and predicted drug resistance in tipranavir. (TIF)

S1 File Source code of Partial Least Squares. (TXT)
