# Supplementary material for: Prediction of HIV drug resistance based on the 3D protein structure: Proposal of molecular field mapping
Source: PLoS One. 2021 Aug 4;16(8):e0255693. doi: 10.1371/journal.pone.0255693 (PMC8336827; doi:10.1371/journal.pone.0255693)
Supplement: S1 Table — There are several available complex structures with HIV protease variants for each drug. For each protease variant to be subjected to homology modeling, one of the protein structures listed was selected as a template according to the similarity of primary amino acid sequences. (DOCX) [file pone.0255693.s001.docx]

**S1 Table. Template proteins for homology modeling of HIV protease variants.**

| Drug | Template (PDB ID) | | | |
| --- | --- | --- | --- | --- |
| Atazanavir | **3EKW** | 3KEY |  |  |
| Darunavir | **3JVY** | 3JW2 |  |  |
| Fosamprenavir | **3NU9** | 3NUJ | 3NUO |  |
| Indinavir | **1SDT** | 1SDV | 1SGU | 1HSG |
| Lopinavir | **6DJ1** | 6DJ2 | 1MUI |  |
| Nelfinavir | **2PYM** | 2PYN | 2Q63 | 2Q64 |
| Saquinavir | **3D1Y** |  |  |  |
| Tipranavir | **2O4P** | 2O4N |  |  |

There are several available complex structures with HIV protease variants for each drug. For each protease variant to be subjected to homology modeling, one of the protein structures listed was selected as a template according to the similarity of primary amino acid sequences.
