# Supplementary material for: Prediction of HIV drug resistance based on the 3D protein structure: Proposal of molecular field mapping
Source: PLoS One. 2021 Aug 4;16(8):e0255693. doi: 10.1371/journal.pone.0255693 (PMC8336827; doi:10.1371/journal.pone.0255693)
Supplement: S2 Table — a) Grid sizes were set to embed an overlaid aggregate of all HIV protease variants. b) Numbers of steric and electrostatic potential energies. (DOCX) [file pone.0255693.s002.docx]

**S2 Table. Number of molecular field energies selected as a feature of machine learning.**

| Drug | Grid size^a)^  (Å^3^) | Before feature selection | | | After feature selection | | | |
| --- | --- | --- | --- | --- | --- | --- | --- | --- |
|  |  | Steric^b)^ | Electrostatic^b)^ | Total | | Steric^b)^ | Electrostatic^b)^ | Total |
| Atazanavir | 39 × 27 × 28 | 29,484 | 29,484 | 58,968 | | 1,223 | 2,728 | 3,951 |
| Darunavir | 27 × 35 × 36 | 34,020 | 34,020 | 68,040 | | 1,094 | 3,443 | 4,537 |
| Fosamprenavir | 27 × 34 × 35 | 32,130 | 32,130 | 64,260 | | 1,210 | 3,268 | 4,478 |
| Indinavir | 34 × 27 × 36 | 33,048 | 33,048 | 66,096 | | 1,121 | 2,549 | 3,670 |
| Lopinavir | 27 × 34 × 36 | 33,048 | 33,048 | 66,096 | | 1,096 | 2,664 | 3,760 |
| Nelfinavir | 30 × 28 × 40 | 33,600 | 33,600 | 67,200 | | 1,199 | 2,344 | 3,543 |
| Saquinavir | 27 × 35 × 35 | 33,075 | 33,075 | 66,150 | | 1,107 | 2,362 | 3,469 |
| Tipranavir | 26 × 35 × 36 | 32,760 | 32,760 | 65,520 | | 1,127 | 3,285 | 4,412 |

a) Grid sizes were set to embed an overlaid aggregate of all HIV protease variants.

b) Numbers of steric and electrostatic potential energies.
