# Supplementary material for: Prediction of HIV drug resistance based on the 3D protein structure: Proposal of molecular field mapping
Source: PLoS One. 2021 Aug 4;16(8):e0255693. doi: 10.1371/journal.pone.0255693 (PMC8336827; doi:10.1371/journal.pone.0255693)
Supplement: S3 Table — (DOCX) [file pone.0255693.s003.docx]

**S3 Table. Sample size of training and external test datasets for each drug**

| Drug | Number of training dataset | | Number of external test dataset | | Total |
| --- | --- | --- | --- | --- | --- |
|  | Complete sequencing samples | Incomplete sequencing samples | Complete sequencing samples | Incomplete sequencing samples |  |
| Atazanavir | 370 | 324.09 | 93 | 83.89 | 870.97 |
| Darunavir | 211 | 219.54 | 53 | 55.96 | 539.50 |
| Fosamprenavir | 580 | 457.02 | 146 | 124.61 | 1307.63 |
| Indinavir | 607 | 463.50 | 152 | 134.35 | 1356.86 |
| Lopinavir | 480 | 436.15 | 120 | 99.98 | 1136.13 |
| Nelfinavir | 624 | 484.50 | 157 | 129.17 | 1394.68 |
| Saquinavir | 606 | 479.89 | 152 | 113.05 | 1350.94 |
| Tipranavir | 241 | 253.14 | 61 | 63.63 | 618.77 |
