# Supplementary material for: Prediction of HIV drug resistance based on the 3D protein structure: Proposal of molecular field mapping
Source: PLoS One. 2021 Aug 4;16(8):e0255693. doi: 10.1371/journal.pone.0255693 (PMC8336827; doi:10.1371/journal.pone.0255693)
Supplement: S4 Table — (DOCX) [file pone.0255693.s004.docx]

**S4 Table. Ranges of hyperparameters in model optimization.**

| LightGBM | |  | Random Forest Regression | |  | Support Vector Regression | |
| --- | --- | --- | --- | --- | --- | --- | --- |
| num_leaves | 2 ~ 60 |  | bootstrap | true or false |  | kernel | linear or rbf |
| n_estimators | 2 ~ 60 |  | max_depth | 5 ~ 150 |  | C | 0 ~ 4 |
| bagging_fraction | 0 ~ 1 |  | max_features | auto or sqrt |  | epsilon | 0 ~1 |
|  |  |  | min_samples_leaf | 1 ~ 10 |  |  |  |
|  |  |  | min_samples_split | 2 ~ 10 |  |  |  |
|  |  |  | n_estimators | 5 ~ 150 |  |  |  |
