# Supplementary material for: Prediction of HIV drug resistance based on the 3D protein structure: Proposal of molecular field mapping
Source: PLoS One. 2021 Aug 4;16(8):e0255693. doi: 10.1371/journal.pone.0255693 (PMC8336827; doi:10.1371/journal.pone.0255693)
Supplement: S5 Table — a) Abbreviations: ATV, atazanavir; DRV, darunavir; FPV, fosamprenavir; IDV, indinavir; LPV, lopinavir; NFV, nelfinavir; SQV, saquinavir; TPV, tipranavir. (DOCX) [file pone.0255693.s005.docx]

**S5 Table. Optimized hyperparameters of LightGBM (LGBM), Random Forest Regression (RF), Support Vector Regression (SVR), and Partial Least Squares (PLS) models for each drug.**

| Model | Hyperparameter | Drug^a)^ | | | | | | | |
| --- | --- | --- | --- | --- | --- | --- | --- | --- | --- |
|  |  | ATV | DRV | FPV | IDV | LPV | NFV | SQV | TPV |
| LGBM | num_leaves | 9 | 12 | 12 | 13 | 9 | 17 | 8 | 20 |
|  | n_estimators | 56 | 60 | 58 | 60 | 60 | 60 | 53 | 52 |
|  | bagging_fraction | 0.19 | 0.482 | 0.012 | 0.993 | 0.98 | 0.293 | 0.779 | 0.996 |
|  |  |  |  |  |  |  |  |  |  |
| RF | bootstrap | true | true | true | true | true | true | true | true |
|  | max_depth | 864 | 345 | 998 | 178 | 756 | 386 | 453 | 624 |
|  | max_features | auto | auto | auto | auto | auto | auto | auto | auto |
|  | min_samples_leaf | 7 | 2 | 2 | 2 | 8 | 3 | 3 | 6 |
|  | min_samples_split | 10 | 10 | 10 | 10 | 10 | 10 | 7 | 10 |
|  | n_estimators | 102 | 108 | 150 | 50 | 105 | 12 | 11 | 6 |
|  |  |  |  |  |  |  |  |  |  |
| SVR | kernel | linear | linear | linear | rbf | linear | linear | linear | linear |
|  | C | 0.002 | 3.053 | 1.075 | 3.461 | 0.002 | 3.361 | 0.012 | 0.861 |
|  | epsilon | 0.328 | 0.256 | 0.477 | 0.004 | 0.332 | 0.467 | 0.348 | 0.34 |
|  |  |  |  |  |  |  |  |  |  |
| PLS | number of principal components | 41 | 111 | 34 | 60 | 98 | 113 | 28 | 112 |

a) Abbreviations: ATV, atazanavir; DRV, darunavir; FPV, fosamprenavir; IDV, indinavir; LPV, lopinavir; NFV, nelfinavir; SQV, saquinavir; TPV, tipranavir.
