# Supplementary material for: Prediction of HIV drug resistance based on the 3D protein structure: Proposal of molecular field mapping
Source: PLoS One. 2021 Aug 4;16(8):e0255693. doi: 10.1371/journal.pone.0255693 (PMC8336827; doi:10.1371/journal.pone.0255693)
Supplement: S6 Table — (DOCX) [file pone.0255693.s006.docx]

**S6 Table. Weighted determination coefficients for prediction in training dataset (*R*^2^).**

| Drug | LightGBM | Random Forest Regression | Support Vector Regression | Partial Least Squares |
| --- | --- | --- | --- | --- |
| Atazanavir | 0.948 | 0.954 | 0.888 | 0.853 |
| Darunavir | 0.981 | 0.951 | 0.911 | 0.901 |
| Fosamprenavir | 0.929 | 0.946 | 0.822 | 0.778 |
| Indinavir | 0.951 | 0.958 | 0.986 | 0.839 |
| Lopinavir | 0.952 | 0.968 | 0.921 | 0.896 |
| Nelfinavir | 0.957 | 0.925 | 0.831 | 0.819 |
| Saquinavir | 0.905 | 0.945 | 0.892 | 0.748 |
| Tipranavir | 0.975 | 0.827 | 0.781 | 0.803 |
