# Supplementary material for: Prediction of HIV drug resistance based on the 3D protein structure: Proposal of molecular field mapping
Source: PLoS One. 2021 Aug 4;16(8):e0255693. doi: 10.1371/journal.pone.0255693 (PMC8336827; doi:10.1371/journal.pone.0255693)
Supplement: S7 Table — a) Data represents mean ± SEM. (DOCX) [file pone.0255693.s007.docx]

**S7 Table. Weighted determination coefficients for prediction of 3-fold cross-validation ^a)^**

| Drug | LightGBM | Random Forest Regression | Support Vector Regression | Partial Least Squares |
| --- | --- | --- | --- | --- |
| Atazanavir | 0.757±0.016 | 0.763±0.010 | 0.746±0.016 | 0.702±0.017 |
| Darunavir | 0.725±0.017 | 0.749±0.012 | 0.748±0.015 | 0.649±0.018 |
| Fosamprenavir | 0.720±0.014 | 0.712±0.015 | 0.710±0.018 | 0.664±0.015 |
| Indinavir | 0.785±0.009 | 0.783±0.007 | 0.761±0.006 | 0.733±0.012 |
| Lopinavir | 0.836±0.010 | 0.840±0.009 | 0.824±0.010 | 0.786±0.013 |
| Nelfinavir | 0.737±0.012 | 0.685±0.011 | 0.687±0.014 | 0.675±0.013 |
| Saquinavir | 0.773±0.007 | 0.744±0.010 | 0.672±0.014 | 0.734±0.010 |
| Tipranavir | 0.476±0.050 | 0.487±0.030 | 0.501±0.033 | 0.44±0.041 |

a) Data represents mean ± SEM.
