# Supplementary material for: Prediction of HIV drug resistance based on the 3D protein structure: Proposal of molecular field mapping
Source: PLoS One. 2021 Aug 4;16(8):e0255693. doi: 10.1371/journal.pone.0255693 (PMC8336827; doi:10.1371/journal.pone.0255693)
Supplement: S8 Table — a) A: All combinations, considering their probability of occurrence, B: All combinations without considering their probability of occurrence, C: Only the complete sequencing data. b) Mean of three runs. (DOCX) [file pone.0255693.s008.docx]

**S8 Table. Effect of weighting of training data on external prediction in Lopinavir.**

| Model | LightGBM | | |  | Random Forest Regression | | |  | Support Vector Regression | | |  | Partial Least Squares | | |
| --- | --- | --- | --- | --- | --- | --- | --- | --- | --- | --- | --- | --- | --- | --- | --- |
| Training dataset^a)^ | A | B | C |  | A | B | C |  | A | B | C |  | A | B | C |
| R^2^ | 0.842 | 0.811 | 0.836 |  | 0.864 | 0.812 | 0.863 |  | 0.808 | 0.811 | 0.799 |  | 0.86 | 0.799 | 0.827 |
| Computation time (msec)^b)^ | 36.9 | 11.4 | 20.8 |  | 92.0 | 98.4 | 104.0 |  | 3080.9 | 1485.5 | 95.8 |  | 2.4 | 2.6 | 2.5 |

a) A: All combinations, considering their probability of occurrence, B: All combinations without considering their probability of occurrence, C: Only the complete sequencing data.

b) Mean of 3 runs.
