# Supplementary material for: Prediction of HIV drug resistance based on the 3D protein structure: Proposal of molecular field mapping
Source: PLoS One. 2021 Aug 4;16(8):e0255693. doi: 10.1371/journal.pone.0255693 (PMC8336827; doi:10.1371/journal.pone.0255693)
Supplement: S9 Table — a) Goodness of classification was evaluated upon defining the threshold as a fold change of 3.5. b) Abbreviations: TPR, true positive ratio; TNR, true negative ratio; FPR, false positive ratio; FNR, false negative ratio; AUC, area under the ROC curve. (DOCX) [file pone.0255693.s009.docx]

**S9 Table. Goodness of classification of Random Forest Regression, Support Vector Regression, and Partial Least Squares models^a)^**

|  | Drug | Accuracy | Precision | TPR^b)^ | TNR^b)^ | FPR^b)^ | FNR^b)^ | AUC^b)^ | F1 score |
| --- | --- | --- | --- | --- | --- | --- | --- | --- | --- |
| Random Forest Regression | Atazanavir | 0.894 | 0.840 | 0.961 | 0.833 | 0.167 | 0.039 | 0.897 | 0.897 |
|  | Darunavir | 0.921 | 0.889 | 0.701 | 0.977 | 0.023 | 0.299 | 0.839 | 0.784 |
|  | Fosamprenavir | 0.869 | 0.744 | 0.901 | 0.855 | 0.145 | 0.099 | 0.878 | 0.815 |
|  | Indinavir | 0.905 | 0.847 | 0.934 | 0.885 | 0.115 | 0.066 | 0.909 | 0.888 |
|  | Lopinavir | 0.910 | 0.878 | 0.940 | 0.884 | 0.116 | 0.060 | 0.912 | 0.908 |
|  | Nelfinavir | 0.899 | 0.873 | 0.941 | 0.854 | 0.146 | 0.059 | 0.898 | 0.906 |
|  | Saquinavir | 0.904 | 0.807 | 0.957 | 0.875 | 0.125 | 0.043 | 0.916 | 0.876 |
|  | Tipranavir | 0.868 | 0.624 | 0.405 | 0.954 | 0.046 | 0.595 | 0.680 | 0.491 |
|  |  |  |  |  |  |  |  |  |  |
| Support Vector Regression | Atazanavir | 0.924 | 0.881 | 0.973 | 0.880 | 0.120 | 0.027 | 0.926 | 0.924 |
|  | Darunavir | 0.954 | 0.900 | 0.874 | 0.975 | 0.025 | 0.126 | 0.925 | 0.887 |
|  | Fosamprenavir | 0.879 | 0.800 | 0.830 | 0.903 | 0.097 | 0.170 | 0.866 | 0.814 |
|  | Indinavir | 0.935 | 0.906 | 0.937 | 0.934 | 0.066 | 0.063 | 0.935 | 0.921 |
|  | Lopinavir | 0.929 | 0.894 | 0.964 | 0.898 | 0.102 | 0.036 | 0.931 | 0.928 |
|  | Nelfinavir | 0.870 | 0.850 | 0.909 | 0.828 | 0.172 | 0.091 | 0.868 | 0.878 |
|  | Saquinavir | 0.894 | 0.802 | 0.930 | 0.875 | 0.125 | 0.070 | 0.903 | 0.861 |
|  | Tipranavir | 0.878 | 0.588 | 0.755 | 0.901 | 0.099 | 0.245 | 0.828 | 0.661 |
|  |  |  |  |  |  |  |  |  |  |
| Partial Least Squares | Atazanavir | 0.918 | 0.863 | 0.985 | 0.857 | 0.143 | 0.015 | 0.921 | 0.920 |
|  | Darunavir | 0.897 | 0.786 | 0.683 | 0.952 | 0.048 | 0.317 | 0.818 | 0.731 |
|  | Fosamprenavir | 0.862 | 0.777 | 0.797 | 0.893 | 0.107 | 0.203 | 0.845 | 0.787 |
|  | Indinavir | 0.911 | 0.880 | 0.904 | 0.916 | 0.084 | 0.096 | 0.910 | 0.892 |
|  | Lopinavir | 0.914 | 0.888 | 0.936 | 0.894 | 0.106 | 0.064 | 0.915 | 0.911 |
|  | Nelfinavir | 0.897 | 0.871 | 0.941 | 0.850 | 0.150 | 0.059 | 0.896 | 0.904 |
|  | Saquinavir | 0.871 | 0.780 | 0.882 | 0.865 | 0.135 | 0.118 | 0.873 | 0.828 |
|  | Tipranavir | 0.884 | 0.612 | 0.724 | 0.914 | 0.086 | 0.276 | 0.819 | 0.663 |

a) Goodness of classification was evaluated upon defining the threshold as a fold change of 3.5. b) Abbreviations: TPR, true positive ratio; TNR, true negative ratio; FPR, false positive ratio; FNR, false negative ratio; AUC, area under the ROC curve.
