# Supplementary figures and images for: Prediction of HIV drug resistance based on the 3D protein structure: Proposal of molecular field mapping
Source: PLoS One. 2021 Aug 4;16(8):e0255693. doi: 10.1371/journal.pone.0255693 (PMC8336827; doi:10.1371/journal.pone.0255693)

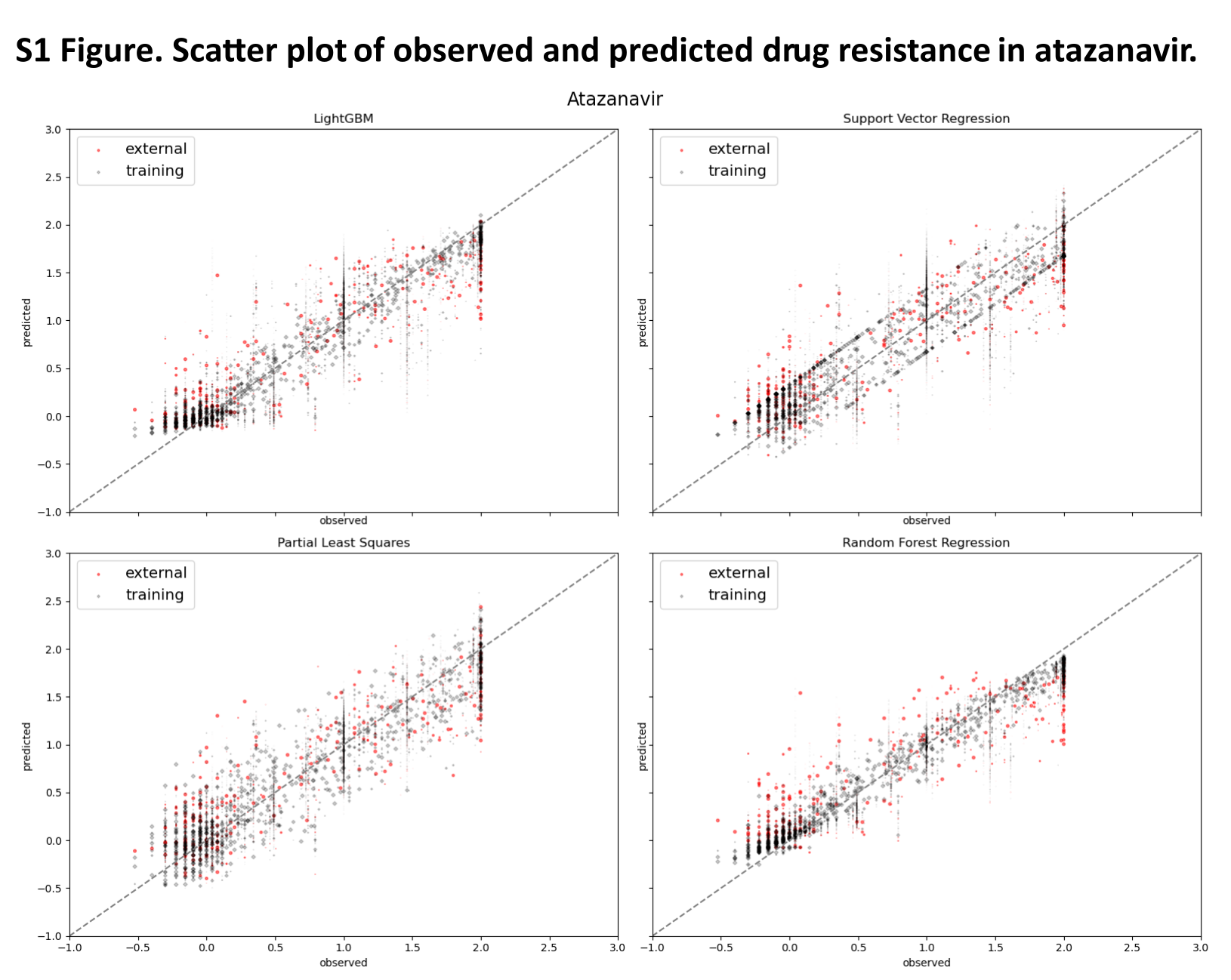

Supplement: S1 Fig — (TIF) [file pone.0255693.s010.tif]

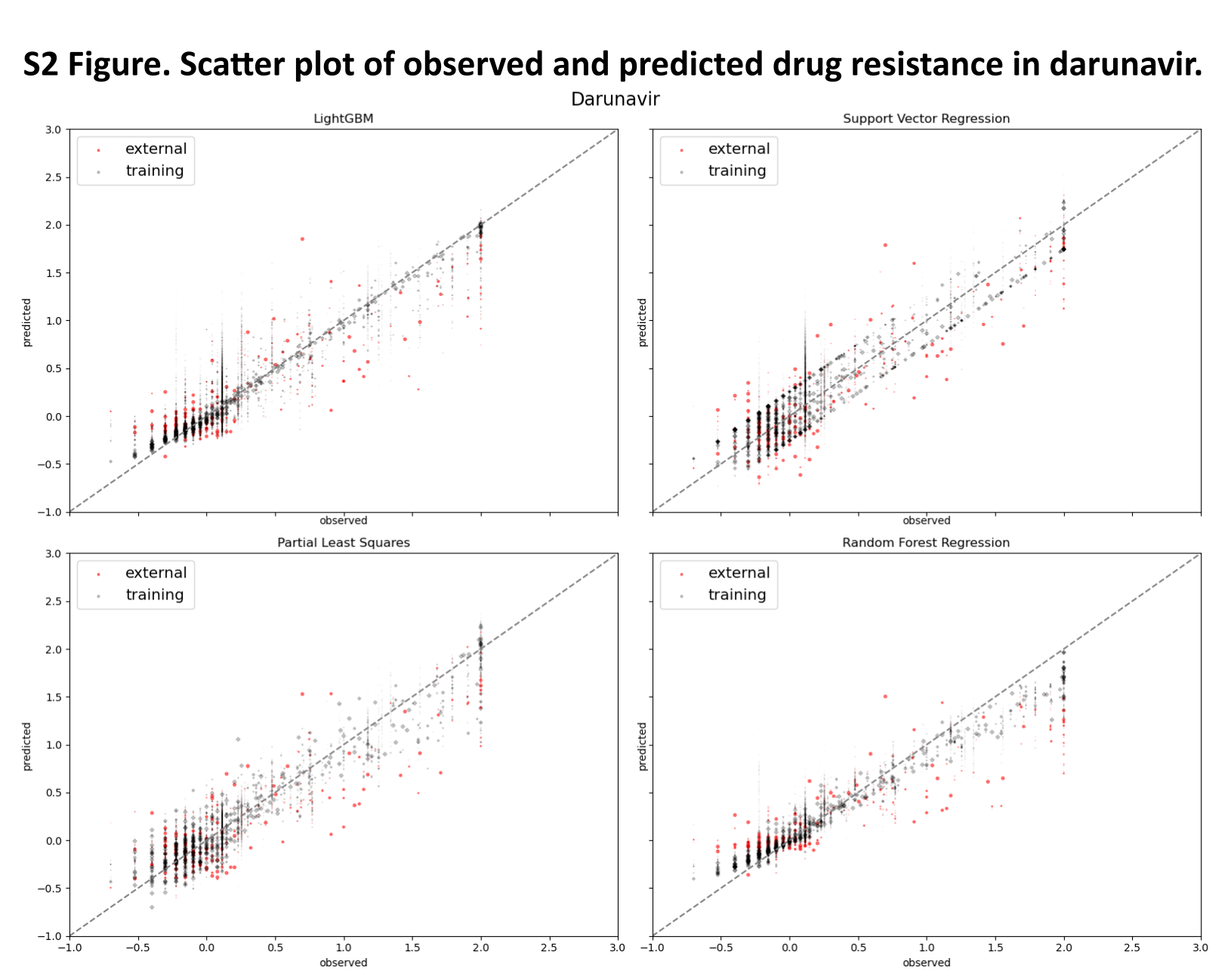

Supplement: S2 Fig — (TIF) [file pone.0255693.s011.tif]

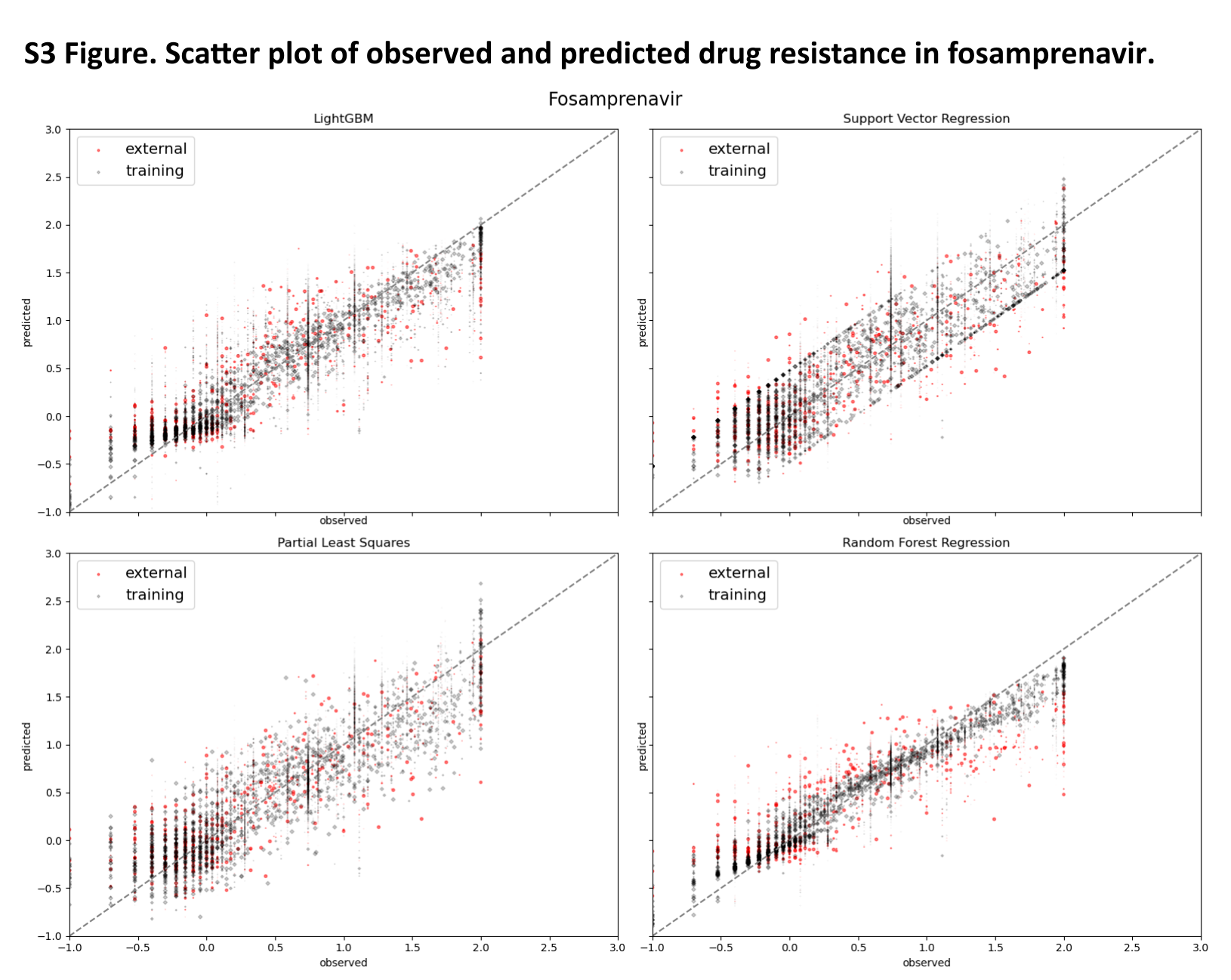

Supplement: S3 Fig — (TIF) [file pone.0255693.s012.tif]

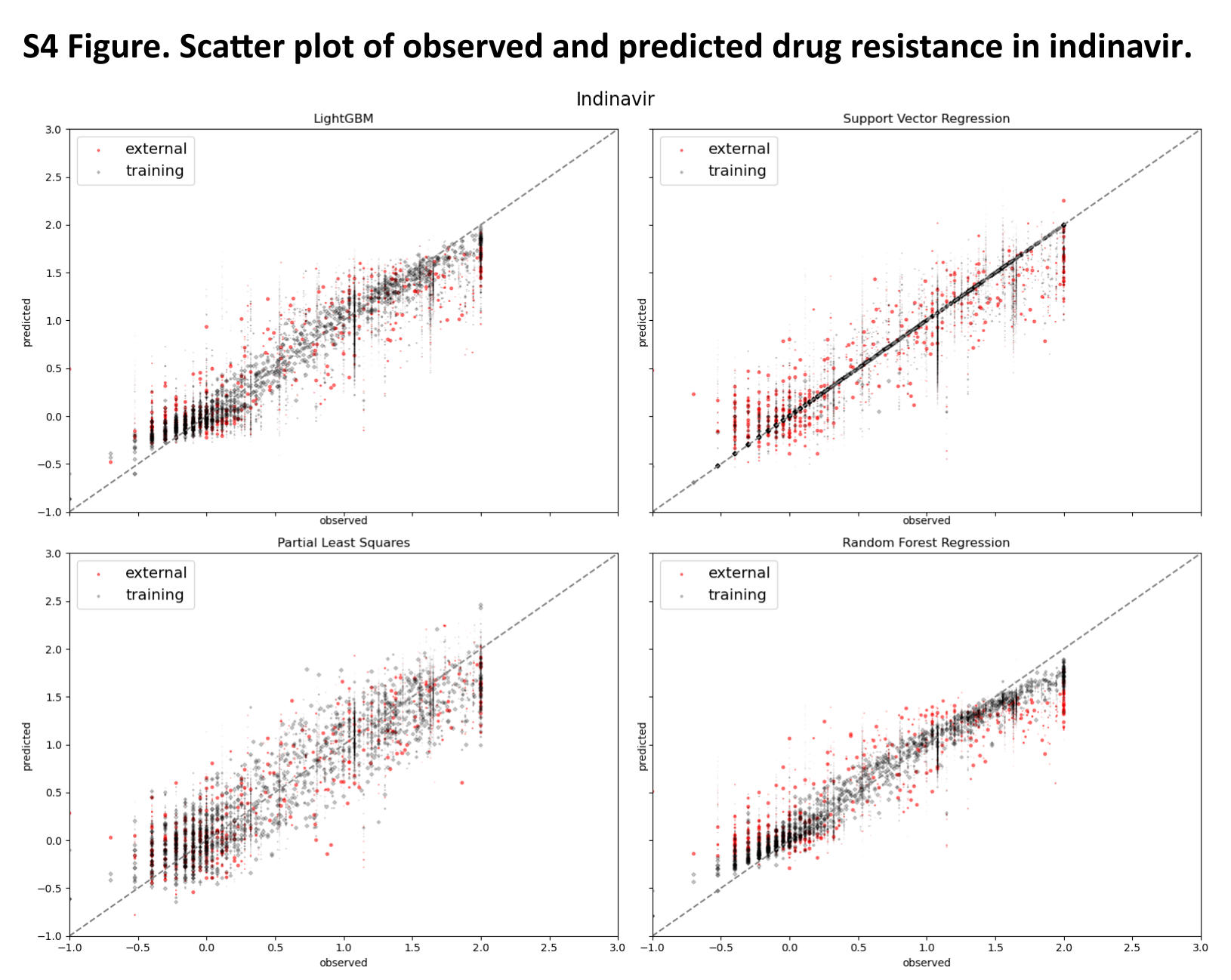

Supplement: S4 Fig — (TIF) [file pone.0255693.s013.tif]

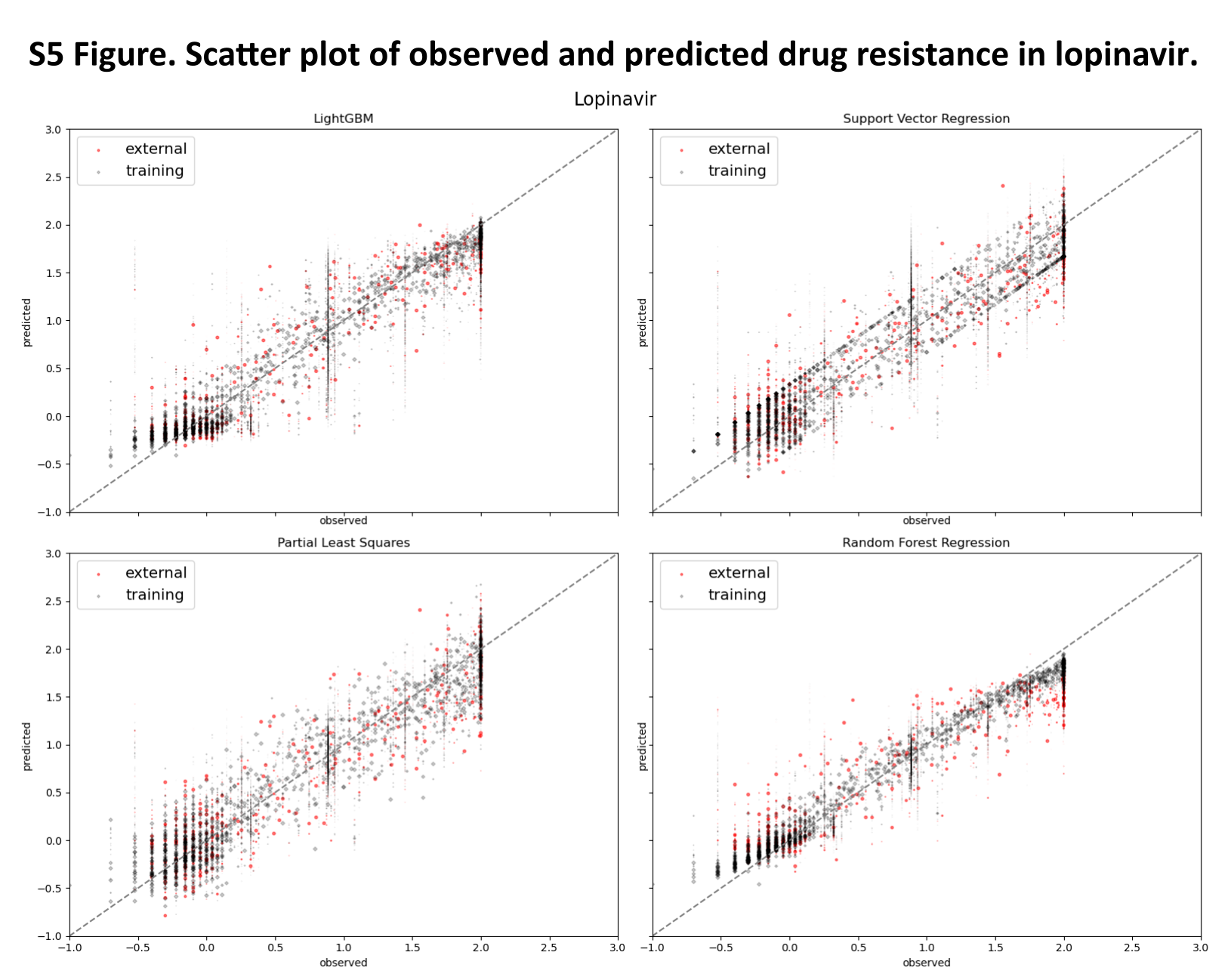

Supplement: S5 Fig — (TIF) [file pone.0255693.s014.tif]

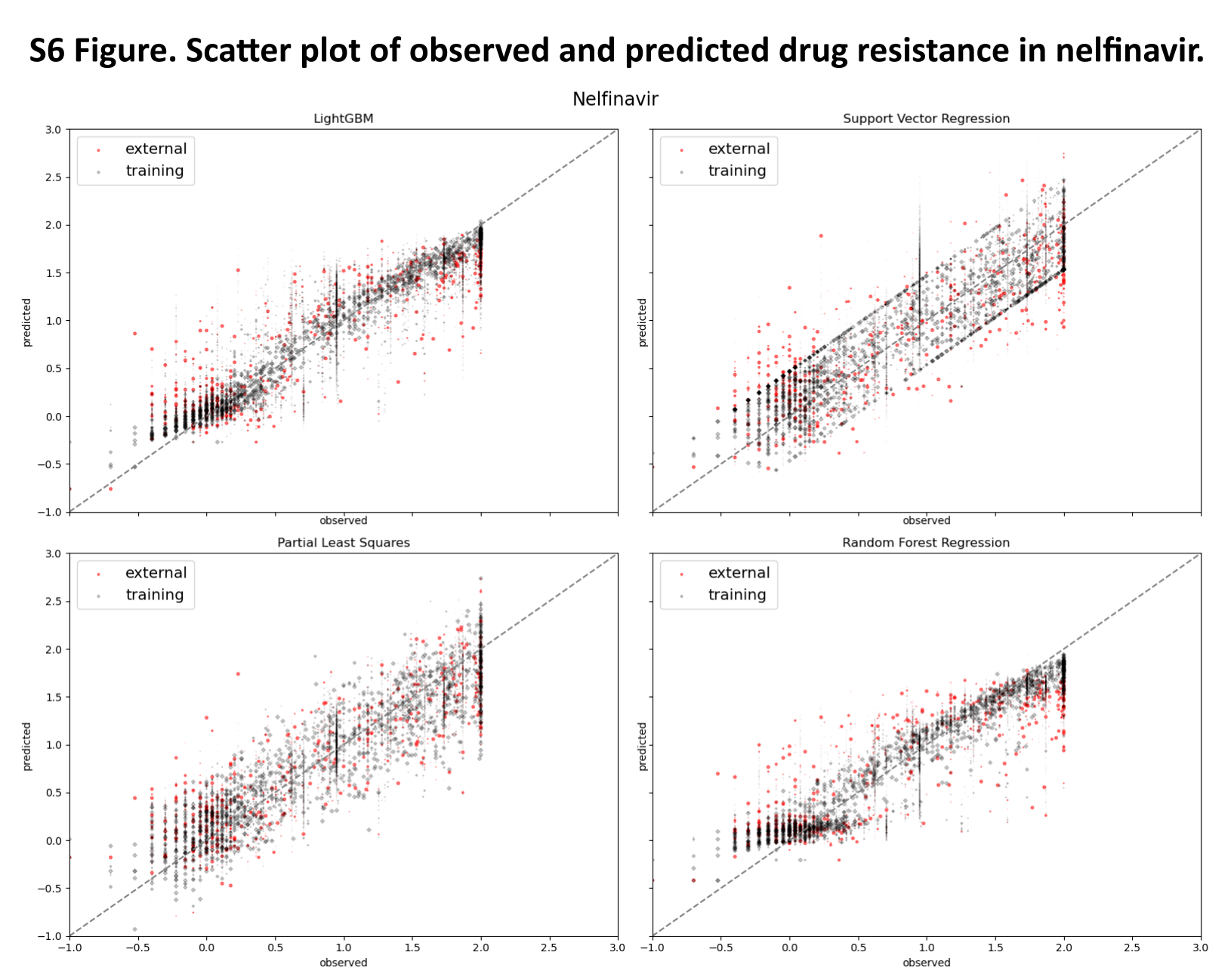

Supplement: S6 Fig — (TIF) [file pone.0255693.s015.tif]

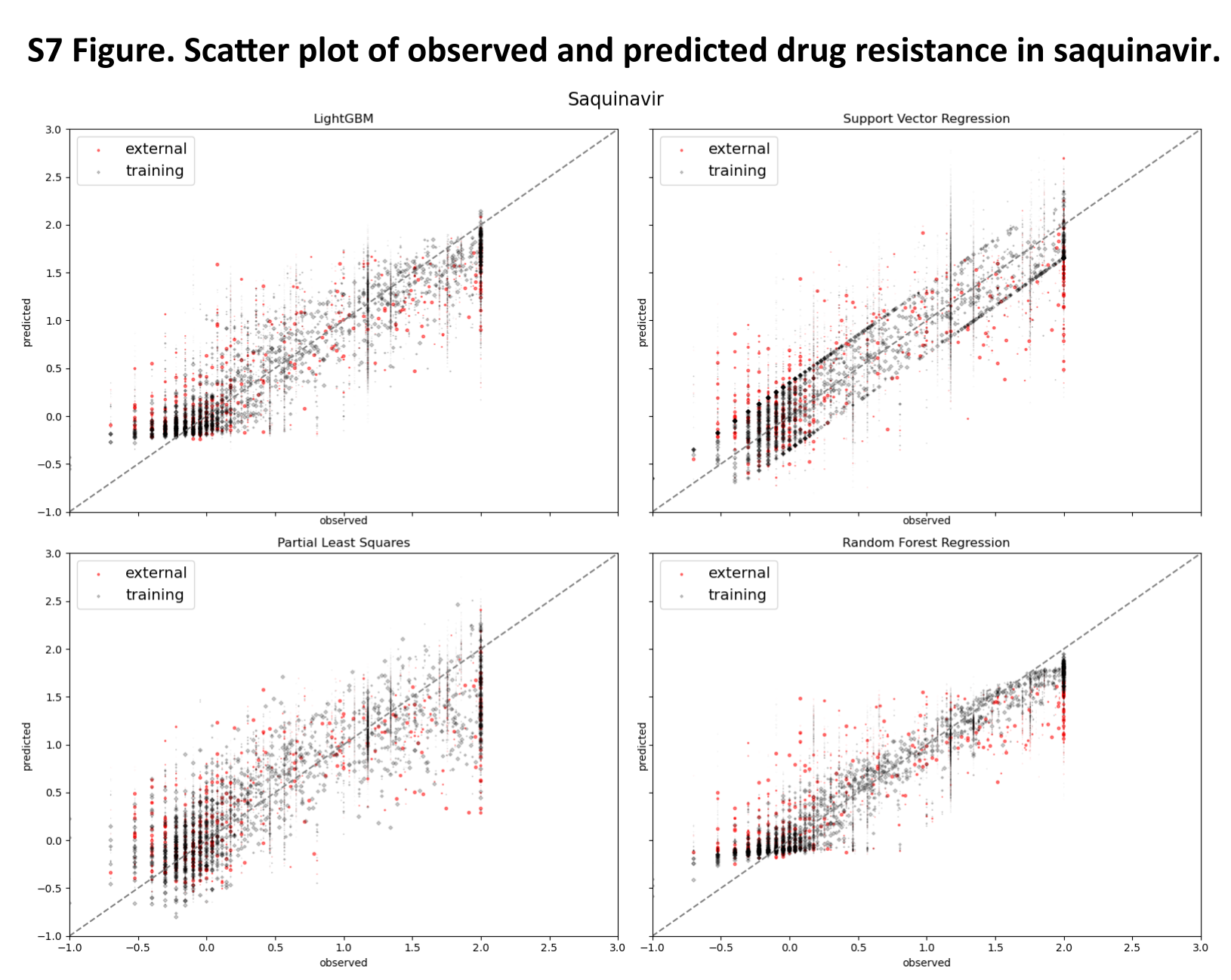

Supplement: S7 Fig — (TIF) [file pone.0255693.s016.tif]

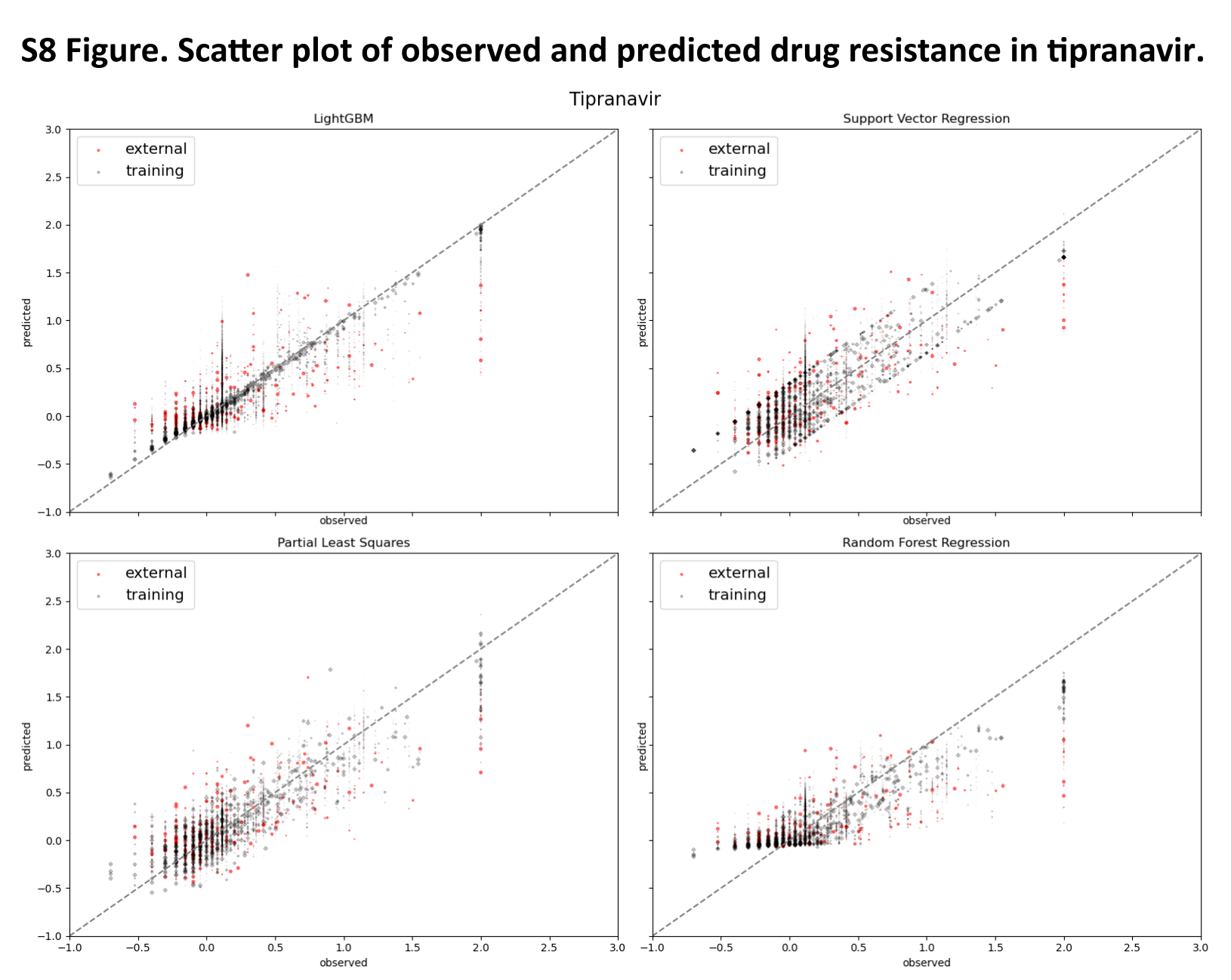

Supplement: S8 Fig — (TIF) [file pone.0255693.s017.tif]
